# Sleeping to fuel the immune system: mammalian sleep and resistance to parasites

**DOI:** 10.1186/1471-2148-9-8

**Published:** 2009-01-09

**Authors:** Mark R Opp

**Affiliations:** 1 Departments of Anesthesiology and Molecular & Integrative Physiology, University of Michigan Medical School, Ann Arbor, MI, USA

## Abstract

Sleep is an enigma. Why animals forgo eating and reproducing, while potentially increasing their risk of predation remains unknown. Although some may question whether all animals sleep, it is clear that all living organisms possess defenses against attack by pathogens. Immune responses of humans and animals are impaired by sleep loss, and responses to immune challenge include altered sleep. Thus, sleep is hypothesized to be a component of the acute phase response to infection and to function in host defense. Examining phylogenetic relationships among sleep parameters, components of the mammalian immune system and resistance to infection may provide insight into the evolution of sleep and lead to a greater appreciation for the role of sleep in host defense.

Sleep is perhaps the greatest enigma in all of biology. Although there have been numerous hypotheses, the question as to the function of sleep remains unanswered. Sleep states are well characterized in mammals and birds, and there is convincing evidence for sleep in animals of "lower" phyla, including invertebrates [1]. Because many characteristics of sleep differ quite dramatically across phyla [2,3], it seems that the lowest common denominator of a core function(s) for sleep must exist at the cellular level [4,5].

Although we may lack an unequivocal functional explanation for sleep, the same is not true for the immune system. Quite simply, the function of the immune system is to keep the organism alive in the face of constant attack by pathogens. The physical and biochemical components of the immune system are integrated into a complex network that is, in many respects, analogous to the networks of cells and transmitter substances that compose the central nervous system. Like sleep, the immune system is phylogenetically ancient. Invertebrates exhibit inflammatory responses to invading pathogens that are functionally equivalent to those of vertebrates [6]. The ability of invertebrates to effectively defend themselves against pathogens is evidenced by their nearly 1 billion years of evolution and the fact they comprise about 95% of the 2 million extant animal species.

The relationship between sleep and the immune system has long been known, but only recently have these interactions been systematically investigated. Investigators have generally used two approaches. In one, human volunteers or laboratory animals are sleep deprived and effects on selected immune parameters determined. The other uses laboratory animals (and in some cases humans) that have been infected with pathogens or challenged with immunomodulators. It is now possible to state that sleep loss alters immune function and that immune challenge alters sleep. What are the mediators of these bi-directional interactions between sleep and the immune system?

Cytokines are the major orchestrators of host defense. Almost twenty of these proteins have been studied to determine effects on sleep. Of these, interleukin-1 (IL-1) and tumor necrosis factor (TNF) have been demonstrated to play a role in the regulation of normal mammalian non rapid eye movement sleep. Data derived from electrophysiological, biochemical and molecular genetic studies demonstrate that antagonizing the IL-1 or the TNF systems reduces spontaneous non rapid eye movement sleep of freely behaving, healthy animals [7,8]. Furthermore, when faced with antigenic challenge, activity of the IL-1 and TNF systems increases and so does non rapid eye movement sleep.

Because sleep loss impairs immune function and immune challenge alters sleep it has been hypothesized that sleep may be considered a component of the acute phase response to infection [9] and functions in host defense [10]. If indeed this is the case, there should be evidence that evolution has favored organisms that optimized the relative investments in sleep and host defense. In this issue, Preston and colleagues [11] ask whether correlations exist between sleep times of mammals and numbers (and types) of white blood cells. Because white blood cells are central to immune responses, these authors used counts as a measure of species investment in the immune system. Their results indicate that across 26 mammalian species those with more total sleep have higher numbers of white blood cells. This positive association of total sleep time with numbers of cells was revealed in four out of the five white blood cell types. Such correlations do not exist for red blood cells, which served as controls because they are derived from the same hematopoietic precursors as white blood cells but have no direct immunological function.

An increase in the number of white blood cells in species that sleep more suggests they should be more immunocompetent. Are there indicators that the association between increased sleep time and white blood cell counts is adaptive? Analysis of sleep times of 12 mammalian species for which data also are available with respect to parasitic load indicates that a 10 hour increase in sleep time is associated with a 24-fold decrease in parasitism. On the basis of this intriguing relationship, Preston et al., [11] conclude that species that have evolved longer sleep times have been able to effectively increase their investment in host defense and reduce the impact of parasites.

Results of this study demonstrate for the first time a correlation between phylogenetic sleep parameters and measures of immune investment. Preston et al., hypothesize that sleep fuels the immune system and they demonstrate increased resistance to parasites in mammalian species that sleep more. But how is resistance to parasites conferred? And what does parasite resistance of mammals suggest about possible evolutionary relationships between sleep and the immune system?

Although physical barriers are the first line of defense against pathogens, increased numbers of white blood cells suggest that what may be important in combating parasites is not just "keeping them out", but the response that is mounted once the pathogen invades and is detected by the host [12-14]. Is it possible that it was host defense responses to antigenic challenge from multiple microbial pathogens that selected for greater numbers of white blood cells and subsequent evolution of sleep rather than increased sleep time leading to greater immunocompetence?

Although they have evolved independently for more than 500 million years, most models postulate a monophyletic origin for vertebrate and invertebrate immune systems that predates the bifurcation in their evolution. For example, IL-1-, IL-6-, TNF-, and transforming growth factor (TGF)-β-like molecules exist in annelids, mollusks, and arthropods (all protostomes), and in echinoderms and protochordates (deuterostomes). Not only do these cytokines exist in invertebrates, they do so in neural tissue [15-18]. The protostome nematode *Caenorhabditis elegans *exhibits a sleep-like state during larval-stage transitions called lethargus [19]. The only gene thus far implicated in sleep-like behavior in *C. elegans *is **egl-4**, which signals through the Decapentaplegic, BMP-like/transforming growth factor-β pathway. Although we do not know whether activation of innate immune defenses alters the lethargus sleep-like state in *C. elegans*, loss of function of Decapentaplegic, BMP-like-1 does increase sensitivity to infection. Several mutant lines of the deuterostome insect *Drosophila *exhibit short or long sleep [1,20-22], and sleep- and wake-related genes have been identified [23]. Expression profiling demonstrates that about 400 *Drosophila *genes are modulated during microbial infection, including the gene for the NF-κB-like transcription factor **relish**. NF-κB is critical in host defense due to its role in cytokine transcription and, as in rodents, flies that lack **relish **sleep less, and **relish **increases during sleep deprivation [24].

Cytokines directly alter properties of neurons in the mammalian brain [25-27], including those in regions involved in the regulation of sleep [28-32]. Although we do not yet know those neural circuits involved in sleep of invertebrates, discharge rates of invertebrate neurons are altered during inflammation or in response to cytokines [33,34]. Responses to antigenic challenge, independent of rest-activity cycles or metabolic demands, could have led to selection of a variety of processes in which ancestral organisms must have engaged. It is not possible to know if responses to antigenic challenge through the course of evolution shaped complex behavior of animals in modern phyla. In theory however, such responses could have driven the evolution of many behaviors and physiological processes in which cytokines are now implicated, including sleep, thermoregulation, feeding, and locomotion. The study by Preston et al., indicates that sleep – immune interactions may also be viewed within the context of phylogeny, thus providing additional clues in the search for core function(s) of sleep.
